# Inducing Autophagy by Rapamycin Before, but Not After, the Formation of Plaques and Tangles Ameliorates Cognitive Deficits

**DOI:** 10.1371/journal.pone.0025416

**Published:** 2011-09-28

**Authors:** Smita Majumder, Arlan Richardson, Randy Strong, Salvatore Oddo

**Affiliations:** 1 Department of Physiology, University of Texas Health Science Center at San Antonio, San Antonio, Texas, United States of America; 2 The Barshop Institute for Longevity and Aging Studies, University of Texas Health Science Center at San Antonio, San Antonio, Texas, United States of America; 3 Department of Pharmacology, University of Texas Health Science Center at San Antonio, San Antonio, Texas, United States of America; 4 Department of Cellular and Structural Biology, University of Texas Health Science Center at San Antonio, San Antonio, Texas, United States of America; 5 Geriatric Research, Education and Clinical Center and Research Service, South Texas Veterans Health Care System, San Antonio, Texas, United States of America; Emory University, United States of America

## Abstract

Previous studies have shown that inducing autophagy ameliorates early cognitive deficits associated with the build-up of soluble amyloid-β (Aβ). However, the effects of inducing autophagy on plaques and tangles are yet to be determined. While soluble Aβ and tau represent toxic species in Alzheimer's disease (AD) pathogenesis, there is well documented evidence that plaques and tangles also are detrimental to normal brain function. Thus, it is critical to assess the effects of inducing autophagy in an animal model with established plaques and tangles. Here we show that rapamycin, when given prophylactically to 2-month-old 3xTg-AD mice throughout their life, induces autophagy and significantly reduces plaques, tangles and cognitive deficits. In contrast, inducing autophagy in 15-month-old 3xTg-AD mice, which have established plaques and tangles, has no effects on AD-like pathology and cognitive deficits. In conclusion, we show that autophagy induction via rapamycin may represent a valid therapeutic strategy in AD when administered early in the disease progression.

## Introduction

Amyloid-β (Aβ) plaques and neurofibrillary tangles (NFTs) are the two neuropathological hallmarks of Alzheimer's disease (AD [1]). Tau, a microtubule-binding protein, is the main constituent of NFTs. In recent years there has been a growing appreciation for a primary role for the build-up of soluble Aβ and tau in the pathogenesis of AD [2]. As the disease progresses, Aβ and tau aggregate to form plaques and NFTs, which further exacerbate AD-associated cognitive impairments [2]. Indeed, evidence showing that Aβ plaques directly alter normal brain function comes from electrophysiological and imaging studies showing that Aβ plaques deregulate normal neuronal firing and lead to structural and functional disruption of neuronal networks [3],[4]. Therefore, when a potential therapeutic agent is assessed in pre-clinical studies (e.g., using mouse models), it is imperative to consider its concomitant effects not only on soluble Aβ and tau but also on plaques and tangles.

Growing evidence highlights the role of autophagy in several age-dependent neurodegenerative disorders characterized by protein accumulation, including AD [5], [6], [7], [8]. Indeed, the well-documented decrease in autophagy function with age may contribute to the accumulation of proteins in the brain [8], [9]. Autophagy is one of the major intracellular proteolytic systems [10]; two key steps in the autophagy system are the formation of the autophagosomes, which are small double membrane organelles that engulf organelles/proteins to be degraded, and the fusion of the autophagosome with the lysosome, where proteins are degraded [11], [12], [13]. The autophagosome formation is mediated by ubiquitin-like reactions of a series of autophagy related proteins (Atg) [11], [12]. The key role that some Atg proteins play in autophagy induction has been shown by knockout experiments [14], [15].

Aging is the major risk factor for the development of AD but little is known about the interaction between aging and AD pathogenesis. Overwhelming evidence shows that reducing the activity of the mammalian target of rapamycin (mTOR) increases lifespan in a variety of organisms [16], [17], [18], [19], [20], [21]. Toward this end, administration of rapamycin, an mTOR inhibitor, significantly increased lifespan in mice [16]. mTOR is a protein kinase that regulates protein homeostasis by facilitating protein translation and inhibiting autophagy [22]. Previous reports have shown that mTOR is hyperactive in selected neurons in AD brains [23], [24], [25], [26], [27], and we have directly linked mTOR hyperactivity to Aβ accumulation [28]. Furthermore, we showed that rapamycin administration increases autophagy and decreases soluble Aβ and tau in young 3xTg-AD mice [29]. However, the concomitant effects of reducing mTOR signaling by rapamycin on plaques and tangles and on the associated learning and memory deficits have not been addressed nor has it become clear whether rapamycin may affect AD-like pathology in old mice. These are critical questions to answer, especially considering that the cognitive deficits associated with AD become significantly more severe with the progression of the disease and the development of plaques and tangles and that age is the major risk factor for the development of AD.

## Results

**Figure 1:**
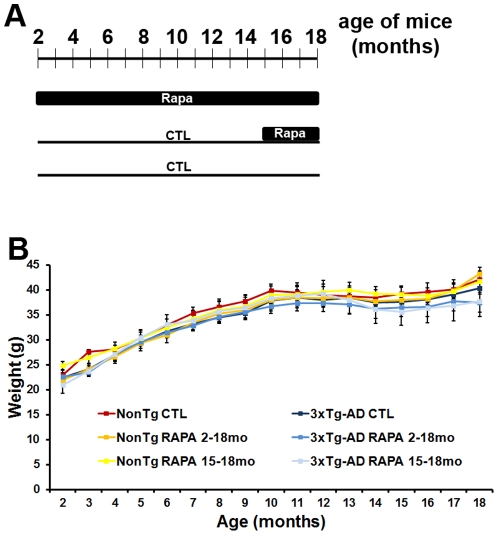
Short- and long-term rapamycin treatments do not cause overt side effects. (**A**) Schematic of the experimental design. 3xTg-AD and NonTg mice were randomly assigned to one of the following groups: (i) 20 mice/genotype fed rapamycin-containing food starting at 2 months of age for 16 months; (ii) 20 mice/genotype fed control diet for the first 15 months of their life after which they were fed rapamycin-containing food for 3 months; (iii) 20 mice/genotype fed control diet throughout their life. All mice were 18 months of age at the end of the treatment. (**B**) All mice gained weight throughout the treatment and no statistically significant differences were found among the groups. Data are presented as means ± SEM.

To determine the effects of rapamycin on AD-like pathology in the 3xTg-AD mice, microencapsulated rapamycin (14 mg/kg) was added to the chow of the following groups of mice: (i) 2-month-old 3xTg-AD and NonTg mice fed rapamycin for 16 months (herein referred to as 3xTg-AD^2–18^ and NonTg^2–18^); (ii) 3xTg-AD and NonTg mice fed the control diet until 15 months of age, after which mice were switched to the rapamycin diet for 3 additional months (herein referred to as 3xTg-AD^15–18^ and NonTg^15–18^); (iii) 3xTg-AD and NonTg mice fed the control diet throughout the experiment (herein referred to as 3xTg-AD^CTL^ and NonTg^CTL^) (Fig. 1A). Overall, we used 120 mice, 20 per genotype per group; all mice were 18 months of age at the end of treatments. Notably, at 2 months of age, the 3xTg-AD mice do not have any apparent neuropathological alterations or learning and memory deficits [30], [31]. In contrast, 15-month-old 3xTg-AD mice have well established plaques and tangles throughout their brains and robust cognitive deficits [30], [31]. Thus, we are positioned to address whether rapamycin can prevent the development of AD-like pathology in the 3xTg-AD mice (by starting the treatment at 2 months of age) or whether it can reverse established plaques, tangles and cognitive deficits (by starting the treatment at 15 months of age). The 3xTg-AD^15-18^ mice were treated for 3 months based on previous studies showing that in young mice 10-12 weeks of rapamycin administration is sufficient to reduce soluble Aβ and tau [29], [32]. The 3xTg-AD^2–18^ mice were treated for 16 months in order to reach the same age as the 3xTg-AD^15–18^ during the behavioral tests and the neuropathological assessments. All 6 groups of mice significantly and equally gained weight over the treatment with no statistically significant differences observed across genotype or treatment group (Fig 1B).

### Early, but not late rapamycin administration ameliorates learning and memory deficits

At the end of the rapamycin administration, mice from all of the groups were 18 months of age. At this age, the 3xTg-AD mice have robust behavioral deficits in cortical- and hippocampal-dependent tasks [31], [33]. To determine the effects of rapamycin on learning and memory, during last 10 days of treatment, mice were tested using two independent behavioral paradigms: the spatial version of the Morris water maze (MWM), a hippocampal-dependent task [34], and the object recognition task, a behavioral task mainly dependent on multiple cortical areas, including the perirhinal cortex [35], [36].

**Figure 2:**
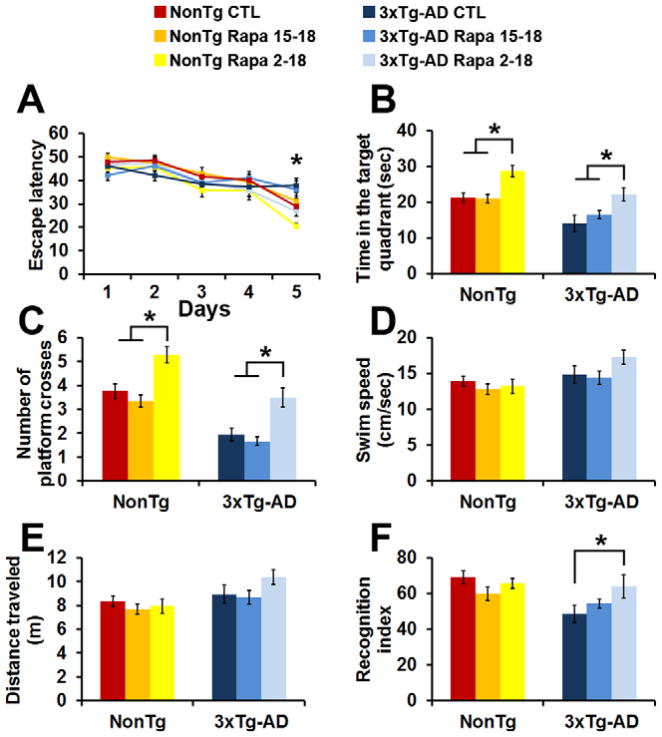
Rapamycin prevents, but does not rescue learning and memory deficits. (**A**) Mice were evaluated in the spatial reference version of the MWM. Mice significantly learned the task over the 5 days of training, as indicated by a reduced time to find the escape platform (F = 36.2; p<0.0001 as calculated by a mixed-model repeated-measures ANOVA). There was also a significant genotype/treatment-day interaction (F = 2.68; p = 0.021). Bonferroni post hoc analysis showed that the NonTg^2–18^ mice learned the task significantly quicker than the NonTg^CTL^ mice. In contrast, the NonTg^15–18^ mice learned as well as the NonTg^CTL^ mice. Similarly, 3 months of rapamycin treatment did not improve learning in the 3xTg-AD mice as the 3xTg-AD^15–18^ mice performed similarly to the 3xTg-AD^CTL^ mice. In contrast, we found that the 3xTg-AD^2–18^ mice learned the task significantly quicker than 3xTg-AD^CTL^ mice and as well as NonTg^CTL^ mice. (**B–C**) Reference memory, measured 24 hours after the last training trials was significantly improved only in the NonTg^2–18^ and 3xTg-AD^2–18^ mice compared to the NonTg^CTL^ and 3xTg-AD^CTL^ mice, respectively. Three months of rapamycin administration, however, did not have any effect on reference memory. (**D–E**) Swimming speed and distance traveled during the probe trials were not significantly different among the 6 groups of mice. (**F**) Mice were also tested using the object recognition task, a cortical-dependent task. One-way ANOVA showed significant changes in the time mice spent exploring the new object across the 6 different groups (Fig. 2F; p = 0.01). Post-hoc analysis showed that short- and long-term rapamycin treatment had no effect on NonTg mice. In contrast, the 3xTg-AD^2–18^ mice performed significantly better than the 3xTg-AD^CTL^ mice. Data are presented as means ± SEM.

During the MWM, mice received 4 training trials per day for 5 consecutive days to find a hidden platform. Their performance was analyzed using a mixed-model, repeated-measures ANOVA, with treatment and genotype as the categorically fixed effects, days as the numeric covariate, animals as the random effect, and escape latency as the dependent variable. We found a significant effect for days (F = 36.2; p<0.0001), indicating that the mice learned the task across sessions (Fig. 2A). More important, we found a significant genotype/treatment-day interaction (F = 2.68; p = 0.021), indicating that one or more of the groups was different from the others (Fig. 2A). To find which group(s) was different from the others, we performed a post hoc test with Bonferroni corrections and compared each of the individual groups to the NonTg mice on the control diet. We found that the NonTg^2–18^ mice performed significantly better than NonTg^CTL^ (p<0.05; escape latency at day 5 was 20.7±1.05 seconds and 29.1±2.7 seconds, respectively). This is consistent with the beneficial effects of rapamycin on mice's health span [37]. In contrast, the escape latency of the NonTg^15–18^ mice was not statistically different from NonTg^CTL^ mice (p<0.05; Fig. 2A). When we analyzed the performance of the 3xTg-AD mice, we found that 3xTg-AD^CTL^ mice performed significantly worse than NonTg^CTL^ mice (p<0.05; escape latency at day 5 was 37.96±2.9 and 29.1±2.7, respectively), which is consistent with previous reports [31]. Furthermore, we found that the 3xTg-AD^15–18^ mice performed similarly to the 3xTg-AD mice on the control diet (Fig. 2A). Most notably, however, we found that when rapamycin was administered for 16 months, starting at 2 months of age, the escape latency of the 3xTg-AD^2–18^ mice was improved, as these mice performed significantly better than the 3xTg-AD^CTL^ mice (p<0.05; escape latency at day 5 was 26.9±2.1 and 37.9±2.9, respectively). In summary, we found that rapamycin, when used prophylactically, significantly improves spatial learning in both 3xTg-AD and NonTg mice (Fig. 2A). In contrast, rapamycin started at 15 months of age has no significant effect on spatial learning in either NonTg or 3xTg-AD mice.

Twenty-four hours after the last training trial, we tested spatial memory by measuring the time mice spent in the target quadrant and the number of platform location crosses over a 60-second probe trial. One-way ANOVA indicated significant changes in the time the mice spent in the target quadrant and the number of platform location crosses (Fig. 2B–C; p = 0.03 and p<0.0001, respectively). A post hoc test with Bonferroni correction showed that the NonTg^2–18^ mice performed significantly better that the NonTg^15–18^ mice and the NonTg^CTL^ mice in both tasks (p<0.05). Similarly, the 3xTg-AD^2–18^ mice performed significantly better than 3xTg-AD^15–18^ and 3xTg-AD^CTL^ mice in both tasks (p<0.05; Fig. 2B–C). Finally, this post hoc analysis also showed that the 3xTg-AD^2–18^ mice performed similarly to the NonTg^CTL^ mice. To determine whether mouse physical performance may account for the changes in spatial learning and memory, we measured the swim speed and the distance mice traveled during the probe trials. One-way ANOVA indicated that both parameters were not significantly different across all 6 groups of mice (Fig. 2D–E). Taken together these findings clearly indicate that when given prophylactically, rapamycin ameliorated spatial learning and memory in both the NonTg and 3xTg-AD mice.

To assess cortical function, mice were also tested in object recognition, which relies mostly on cortical areas, including the perirhinal cortex [35], [36]. This task exploits the natural tendency of mice to explore objects perceived as novel and therefore is less stressful than the MWM [38], [39]. One-way ANOVA indicated significant changes in the time mice spent exploring the new object across the 6 different groups (Fig. 2F; p = 0.01). To find which group(s) was different from the others, we performed a post hoc test with Bonferroni corrections and compared each of the individual groups to each other. We found that the 3xTg-AD^CTL^ mice performed at a chance level and significantly worse than NonTg^CTL^ mice (p<0.05; Fig. 2F). Furthermore, the post hoc analysis indicated that rapamycin did not improve recognition memory in both groups of the rapamycin-treated NonTg mice (Fig. 2F). Similarly, the 3xTg-AD^15–18^ mice performed at a chance level, indicating that this paradigm treatment had no effect on recognition memory (Fig. 2F). In contrast, we found that the 3xTg-AD^2–18^ mice performed significantly better than the 3xTg-AD^CTL^ mice (p<0.05; Fig. 2F), clearly indicating that rapamycin, when given prophylactically improves recognition memory in the 3xTg-AD mice.

### Rapamycin reduces Aβ plaques and neurofibrillary tangles formation

**Figure 3:**
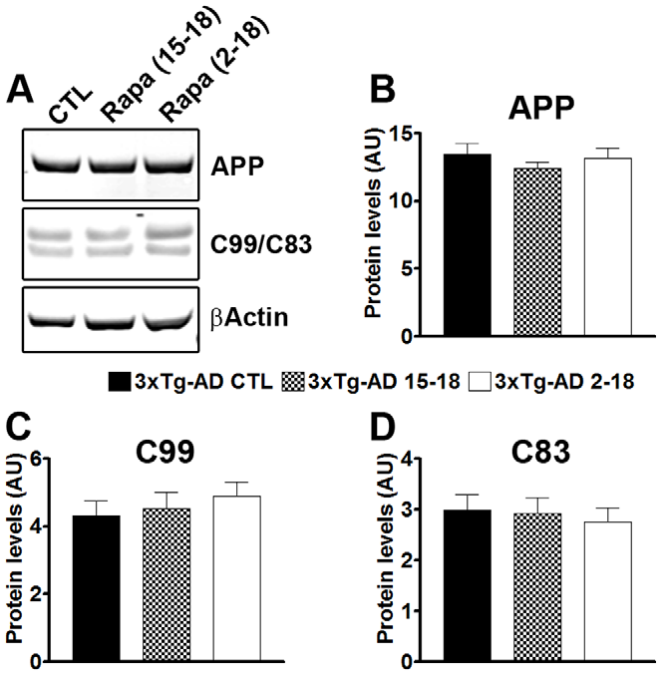
Rapamycin does not change APP processing. (**A**) Representative Western blots of proteins extracted from 3xTg-AD^CTL^, 3xTg-AD^15–18^ and 3xTg-AD^2–18^ mice (n = 8/group) and probed with the indicated antibodies. (**B–C**) Quantitative analysis of the blots showed that rapamycin did not change the steady-state levels of full length APP or its two major C-terminal fragments, C99 and C83. Data are presented as means ± SEM and analyzed by one-way ANOVA.

All mice were 18 months of age when sacrificed, which was immediately following the last behavioral testing. To determine the effects of rapamycin on Aβ pathology, we first assessed whether rapamycin altered APP processing. Toward this end, the levels of human brain APP and its two major C-terminal fragments were measured by Western blot (Fig. 3A). Quantitative analysis of the blots showed that the steady-state levels of the APP transgene were similar across the three groups of 3xTg-AD mice, as indicated by one-way ANOVA (p>0.05). The two major C-terminal fragments of APP are C99 and C83, which are generated from the cleavage of APP by α-secretase and BACE, respectively. Western blot analysis, using an antibody against the C-terminal region of APP, showed that neither fragments were altered by rapamycin (Fig. 3C–D; p>0.05 as determined by one-way ANOVA). Together these results show that APP production is not altered by rapamycin administration.

**Figure 4:**
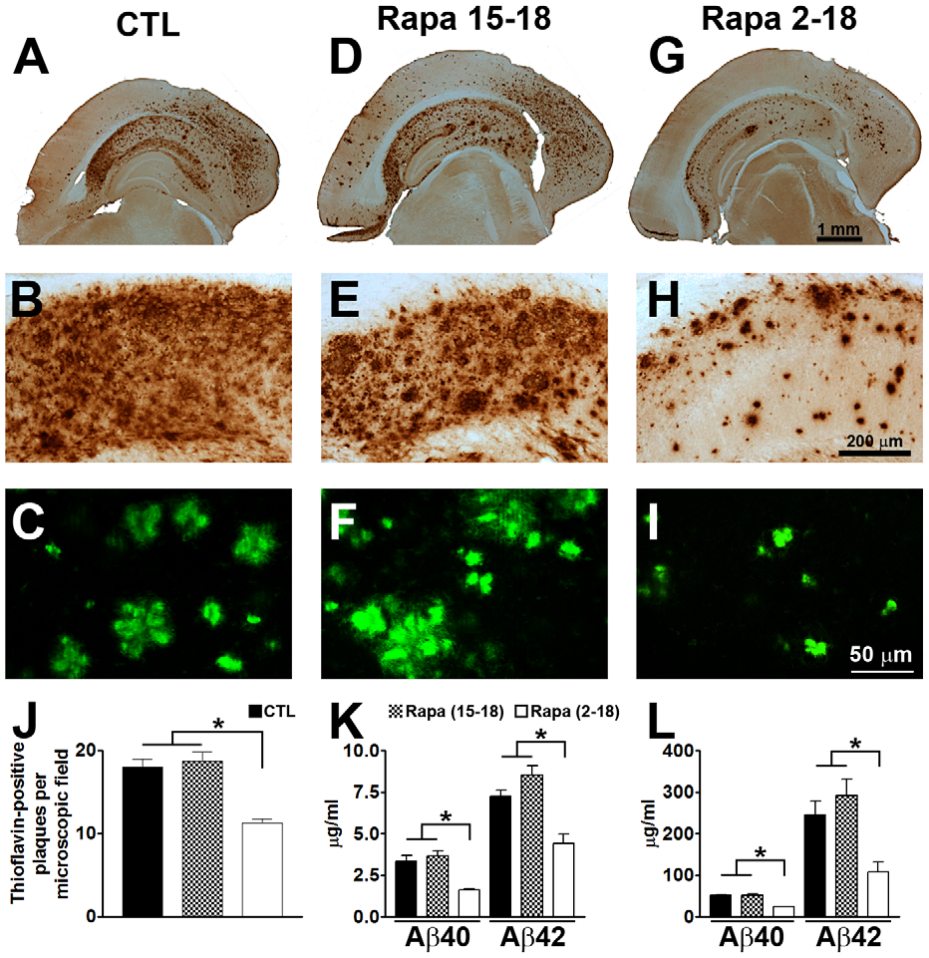
Life-long rapamycin administration reduces Aβ levels and deposition. (**A–I**) Representative sections from brains of 3xTg-AD^CTL^, 3xTg-AD^15–18^ and 3xTg-AD^2–18^ mice (n = 8/group) immunostained with an Aβ-specific antibody (A–B, D–E, G–H) and stained with thioflavin S (C, F, I), clearly show that the 3xTg-AD^2–18^ mice have less diffuse and fibrillar Aβ deposits compared to 3xTg-AD^CTL^ and 3xTg-AD^15–18^ mice. (**J**) Semi-quantitative assessment of the number of thioflavin-positive plaques shows no significant change between 3xTg-AD^CTL^ and 3xTg-AD^15–18^ mice. In contrast, the 3xTg-AD^2–18^ mice have significantly less plaques compared to the other two groups. One-way ANOVA across the three different groups shows that the changes were highly significant (F = 69.65; p<0.0001). Panels B, E and H represent high magnification views of panels A, D and G. (**K–L**) Soluble (K) and insoluble (L) Aβ40 and Aβ42 levels were measured by sandwich ELISA. Consistent with the histological results, compared to 3xTg-AD^CTL^ mice, soluble and insoluble Aβ40 and Aβ42 levels were significantly reduced only in the 3xTg-AD^2–18^ mice (F = 40.50; p<0.0001 for the soluble Aβ levels; F = 22.51 and p<0.0001 for the insoluble Aβ levels). Data are presented as means ± SEM.

We next determined whether plaque load was altered by rapamycin. Toward this end, sections from 3xTg-AD^CTL^, 3xTg-AD^15–18^ and 3xTg-AD^2–18^ mice (n = 8/group) were stained with a widely used anti-Aβ_42_ specific antibody [40], [41], [42], [43]. The 3xTg-AD^CTL^ mice showed a widespread Aβ deposition throughout the brain (Fig. 4A), including the hippocampus (Fig. 4B). Several of these Aβ deposits were also thioflavin S-positive (Fig. 4C), indicating the presence of fibrillar aggregates of Aβ. The Aβ pathology in the 3xTg-AD^15–18^ mice was similar to the 3xTg-AD^CTL^ mice, as indicated by Aβ immunostaining (Fig. 4D–E) and thioflavin (4F). In contrast, we found that Aβ deposition was reduced in the 3xTg-AD^2–18^ mice (Fig. 4G–I). Semi-quantitative analysis showed that the number of the thioflavin-positive plaques in the 3xTg-AD^2–18^ was reduced 52.36% and 55.62% compared to 3xTg-AD^CTL^ and 3xTg-AD^15–18^ mice, respectively (Fig. 4J). This change was highly significant, as indicated by one-way ANOVA analysis (p<0.0001). Bonferroni's post hoc analysis further showed that the number of thioflavin plaques was similar between 3xTg-AD^CTL^ and 3xTg-AD^15–18^ (p>0.05), but both of these groups were significantly different from 3xTg-AD^2–18^ (p<0.001).

To better discriminate between changes in soluble and insoluble Aβ40 and Aβ42 levels, we analyzed brain extracts from treated and untreated 3xTg-AD mice by sandwich ELISA. Consistent with the immunohistochemical analysis, we found that in the 3xTg-AD^2–18^ mice, soluble Aβ40 levels were 52.18% and 56.17% lower compared to 3xTg-AD^CTL^ and 3xTg-AD^15–18^ mice, respectively (Fig. 4K). Similarly, we found that in the 3xTg-AD^2–18^ mice, soluble Aβ42 levels were 38.95% and 48.2% lower compared to 3xTg-AD^CTL^ and 3xTg-AD^15–18^ mice, respectively (Fig. 4K). One-way ANOVA analysis indicated that these changes were significant (p<0.0001). Bonferroni's post hoc analysis indicated that soluble Aβ40 and Aβ42 levels were not significantly different between 3xTg-AD^CTL^ and 3xTg-AD^15–18^ mice (p>0.05). In contrast, soluble Aβ40 and Aβ42 levels were significantly lower in the 3xTg-AD^2–18^ compared to 3xTg-AD^CTL^ and 3xTg-AD^15–18^ mice (for Aβ40, p<0.05; for Aβ42, p<0.001). Consistent with the thioflavin data, we also found that rapamycin significantly decreased insoluble Aβ40 and Aβ42 in the 3xTg-AD^2–18^ mice, while it had no effects on the 3xTg-AD^15–18^ mice (Fig. 4I). Similarly to the soluble Aβ levels, one-way ANOVA showed significant changes in insoluble Aβ40 and Aβ42 levels across the three groups (p<0.0001); Bonferroni's multiple comparison test indicated that the only group in which insoluble Aβ levels were significantly reduced was the 3xTg-AD^2–18^ mice. Together, these data show that rapamycin reduces Aβ plaques formation when administered before plaques are formed. In contrast, under the conditions used here, rapamycin administration has no effect on established plaque pathology. It remains to be determined whether a longer period of rapamycin administration (e.g., from 15 to 24 months of age) may have different effects on Aβ load.

**Figure 5:**
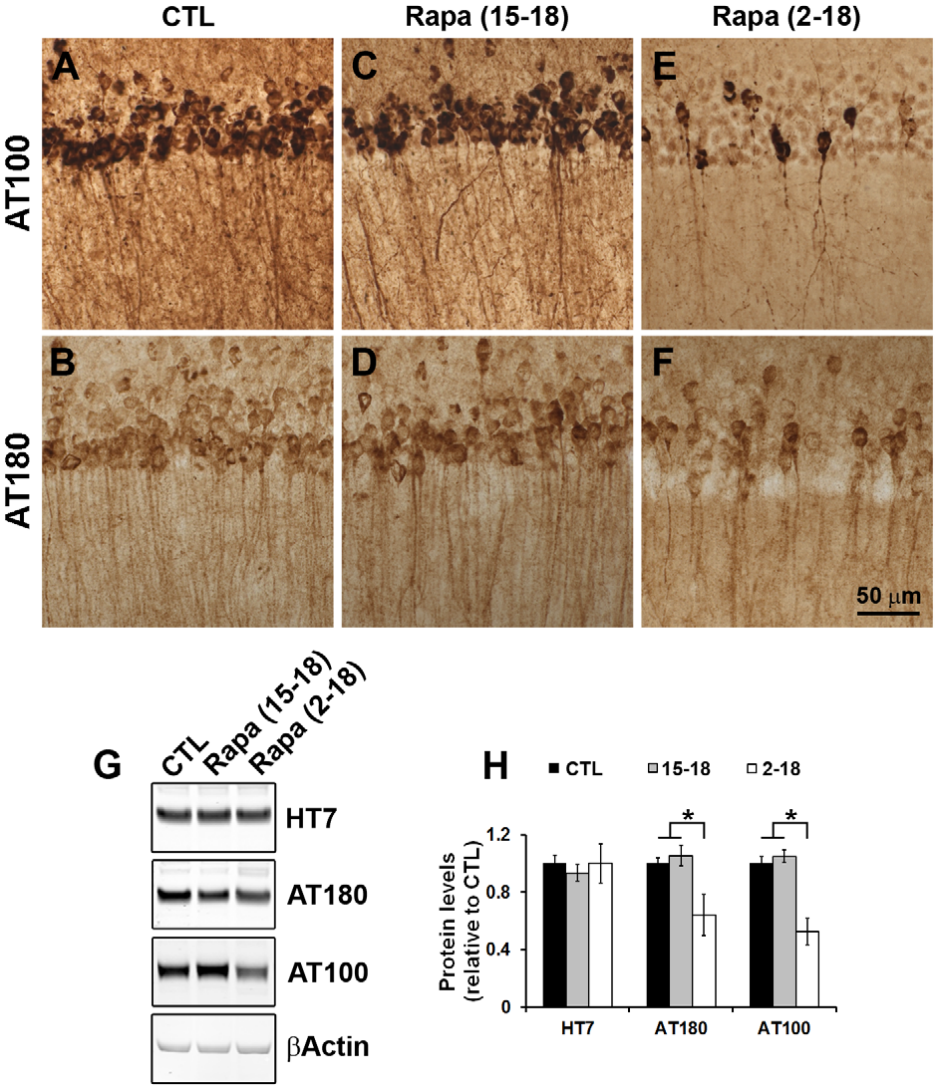
Tau pathology is significantly reduced in 3xTg-AD^2–18^ mice. (**A–F**) Representative sections depicting CA1 pyramidal neurons from brains of 3xTg-AD^CTL^, 3xTg-AD^15–18^ and 3xTg-AD^2–18^ mice (n = 8/group) immunostained with the indicated anti-tau antibodies. Note the reduction of AT100- and AT180-positive neurons in the 3xTg-AD^2–18^ mice compared to 3xTg-AD^CTL^ and 3xTg-AD^15–18^ mice. (**G**) Representative Western blots of proteins extracted from 3xTg-AD^CTL^, 3xTg-AD^15–18^ and 3xTg-AD^2–18^ mice (n = 8/group) and probed with the indicated antibodies. (**H**) Quantitative analysis of the blots shows that rapamycin did not change the steady-state levels of full length tau transgene as measured by the human-specific anti-tau antibody, HT7. In contrast, the levels of tau phosphorylated at the AT100 and AT180 epitopes were significantly reduced in the 3xTg-AD^2–18^ mice compared to the 3xTg-AD^CTL^ and 3xTg-AD^15–18^ mice (F = 5.271 and p = 0.018 for AT100; F = 14.25 and p = 0.0003 for AT180). Data are presented as means ± SEM.

In addition to Aβ pathology, the 3xTg-AD mice develop age-dependent tau pathology [30], [44]. To determine the effects of rapamycin on established neurofibrillary tangles, we stained sections from 3xTg-AD^CTL^, 3xTg-AD^15–18^ and 3xTg-AD^2–18^ mice (n = 8/group) with two different phospho-specific anti-tau antibodies, AT100 and AT180. The former recognizes PHF-tau phosphorylated at Ser212 and Thr214, two amino acids selectively phosphorylated in AD brains [45]; The latter recognizes PHF-tau phosphorylated at Thr231 and Ser235, two amino acids that are physiologically phosphorylated in immature and adult brains, but are strongly hyperphosphorylated in AD brains [46]. In the brains of the 3xTg-AD^CTL^ mice, we found strong immunostaining with both AT100 and AT180 (Fig. 5A–B), which is consistent with previous reports [31], [44]. Rapamycin administration to 3xTg-AD^15–18^ mice had no effect on AT100 and AT180 immunoreactivity (compare Fig. 5 A and C, and B and D). In contrast, we found that the immunoreactivity levels of both epitopes were significantly reduced in the 3xTg-AD^2–18^ mice (Fig. 5E–F).

We next assessed changes in tau pathology using biochemical means. Western blot analysis with a pan human specific tau antibody showed that the steady-state levels of the tau transgene were similar across the three different groups (Fig. 5G–H; p>0.05 as determined by one-way ANOVA). In contrast, we found that the steady state levels of tau phosphorylated at the AT100 and AT180 epitopes were significantly decreased by rapamycin administration (p = 0.018 for AT100 and p = 0.0003 for AT180). Bonferroni's multiple comparison test showed that AT100 and AT180 levels in the 3xTg-AD^2–18^ mice were significantly reduced compared to the 3xTg-AD^CTL^ and 3xTg-AD^15–18^ mice (p<0.05 for AT100 and p<0.01 for AT180). Taken together, these data strongly suggest that life-long rapamycin administration reduces the accumulation of pathological tau, whereas starting rapamycin treatment after tangles are formed has no significant effects on tau pathology.

### Rapamycin reduces microglia activation

**Figure 6:**
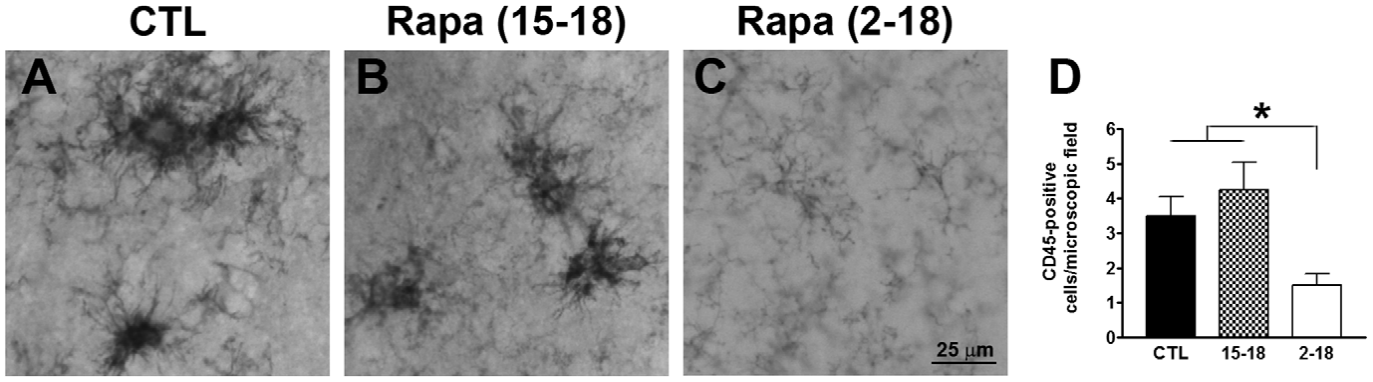
Rapamycin reduced the number of activated microglia. (**A–C**) Representative sections from CA1/subiculum regions of brains from 3xTg-AD^CTL^, 3xTg-AD^15–18^ and 3xTg-AD^2–18^ mice brains (n = 8/group) immunostained with an anti-CD45 antibody. (D) Semi-quantitative analysis showed that the number of activated microglia was significantly different across the three groups as determined by one-way ANOVA (F = 5.7; p = 0.01). Bonferroni's post hoc analysis showed that the number of activated microglia was significantly lower in the 3xTg-AD^2–18^ mice compared to the other two groups. No statistically significant changes were found between 3xTg-AD^CTL^ and 3xTg-AD^15–18^ mice. Data are presented as means ± SEM.

There is some controversy as to the role of brain inflammation in AD pathogenesis [47], [48], [49]. It is clear, however, that microglia activation is an invariable feature of AD pathology [48], [50]. Previous reports have shown that in the 3xTg-AD mice, activated microglia are first detected in the CA1/subiculum region of the hippocampus and correlate with the onset of fibrillar Aβ deposits in the same brain region [51]. To determine whether the reduction in fibrillar Aβ deposits corresponded to a decrease in microglia activation, sections from 3xTg-AD^CTL^, 3xTg-AD^15–18^, 3xTg-AD^2–18^ mice were stained with CD45, a common marker of activated microglia. As expected, 18-month-old 3xTg-AD^CTL^ mice showed clear CD45-positive immunoreactivity throughout the hippocampus (Fig. 6A). When we compared sections from 3xTg-AD^CTL^, 3xTg-AD^15–18^, and 3xTg-AD^2–18^ mice, we found that the number of activated microglia was similar between 3xTg-AD^CTL^ and 3xTg-AD^15–18^ mice (Fig. 6A–B, D). In contrast, we found that the 3xTg-AD^2–18^ mice had a significantly lower number of activated microglia compared to the other two groups of 3xTg-AD mice (Fig. 6A–D; p = 0.01 as determined by one-way ANOVA). Bonferroni post hoc analysis confirmed that the number of activated microglia in 3xTg-AD^2–18^ mice was significantly lower than in the 3xTg-AD^CTL^ and 3xTg-AD^15–18^ mice. In contrast, no difference was detected between 3xTg-AD^CTL^ and 3xTg-AD^15–18^ mice (Fig. 6D). These results highlight a correlation between the reduction in fibrillar Aβ deposits and the number of activated microglia.

### Rapamycin increases autophagy induction in both 3xTg-AD^15–18^ and 3xTg-AD^2–18^ mice

**Figure 7:**
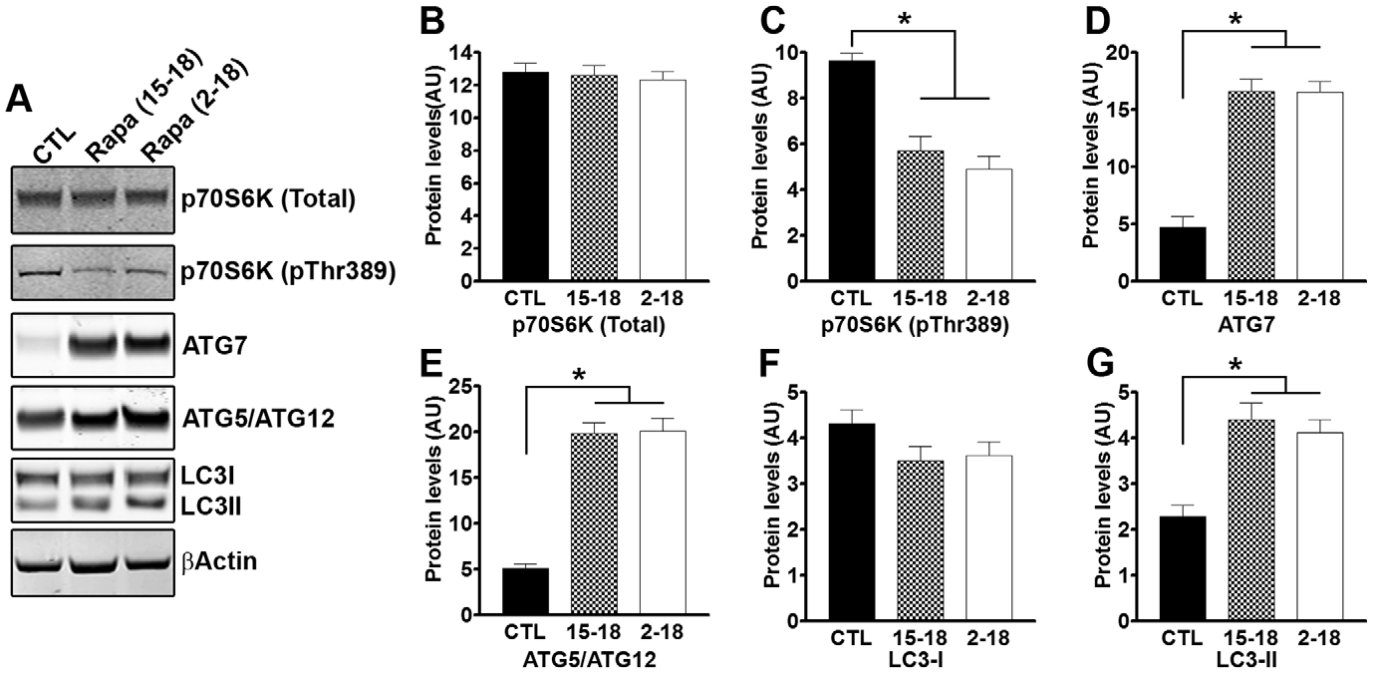
Autophagy is equally induced in 3xTg-AD^2–18^ and 3xTg-AD^15–18^ mice. (**A**) Representative Western blots of proteins extracted from 3xTg-AD^CTL^, 3xTg-AD^15–18^ and 3xTg-AD^2–18^ mice (n = 8/group) and probed with the indicated antibodies. (**B–C**) Quantitative analysis of the blots showed that rapamycin did not change the steady-state levels of total p70S6K. In contrast, the levels of p70S6K phosphorylated at Thr389 (a site directly phosphorylated by mTOR) were significantly changed by rapamycin administration (F = 23.07; p<0.001). Bonferroni's post hoc analysis showed that the levels of p70S6K phosphorylated at Thr389 were not significantly different between 3xTg-AD^2–18^ and 3xTg-AD^15–18^ mice. In contrast, phospho-p70S6K levels in both groups were significantly lower compared to the 3xTg-AD^CTL^ mice (p<0.001). (**D–E**) Similar results were obtained when we quantified the levels of the autophagy-related proteins, Atg7 and Atg5/Atg12. One-way ANOVA showed that there was a group effect for Atg7 (F = 46.92; p<0.0001) and Atg5/Atg12 (F = 64.37; p<0.0001). Post-hoc analysis confirmed that the levels of Atg7 and Atg5/Atg12 were significantly higher in 3xTg-AD^15–18^ and 3xTg-AD^2–18^ mice compared to 3xTg-AD^CTL^ mice, but no significant differences were found between 3xTg-AD^15–18^ and 3xTg-AD^2–18^ mice. (**F–G**) Quantitation of the LC3I/II levels showed that while rapamycin did not alter LC3I levels (F = 2.039; p = 0.1552, as calculated by one-way ANOVA), a significant group effect was found for LC3II levels (F = 14.58; p = 0.0001). Consistent with the Atg levels, the groups responsible for this difference were the 3xTg-AD^15–18^ and 3xTg-AD^2–18^ mice, which showed significantly higher LC3II levels compared to 3xTg-AD^CTL^ mice, as determined by Bonferroni's post-hoc test (p<0.001). The levels of LC3II were not significantly different between the 3xTg-AD^15–18^ and 3xTg-AD^2–18^ mice. Data are presented as means ± SEM.

Rapamycin is a well-known mTOR inhibitor [52]. mTOR activity is routinely assessed by measuring the steady-state levels of downstream targets directly phosphorylated by mTOR. Along these lines, p70S6K is a protein kinase directly phosphorylated by mTOR at threonine 389. Indeed, a large body of evidence shows that the levels of p70S6K phosphorylated at threonine 389 (p70S6K-Thr389) strongly and consistently correlate with mTOR activity [53], [54], [55], [56]. To better understand the molecular mechanisms underlying the rapamycin-mediated decrease in Aβ and tau pathology, we first measured the steady-state levels of total and phosphorylated p70S6K by Western blot. We found that while the total levels of p70S6K were similar among 3xTg-AD^CTL^, 3xTg-AD^15–18^, 3xTg-AD^2–18^ mice (Fig. 7A–B), the levels of p70S6K-Thr389 were significantly decreased in the two rapamycin-treated groups (Fig. 7A, C; p<0.0001 as assessed by one-way ANOVA). Bonferroni's post hoc analysis indicated the 3xTg-AD^15–18^ and the 3xTg-AD^2–18^ mice were both significantly different from 3xTg-AD^CTL^ (p<0.001), but not from each other (Fig. 7C). These data indicate that mTOR signaling is decreased in the brain of rapamycin-treated 3xTg-AD mice and are consistent with previous reports showing that rapamycin does cross the blood brain barrier [52], [57], [58], [59], [60].

mTOR is a negative regulator of autophagy induction, which is one of the two major catabolic processes utilized by cells for protein turnover [61], [62], [63]. Autophagy induction occurs via the activation of a series of autophagy related proteins (Atg), which lead to formation of autophagosomes through a cascade of reactions resembling the ubiquitin conjugation system [11], [13], [64]. Atg5 and Atg7 are two autophagy-related proteins, both of which are necessary for autophagy induction [11], [13]. We initially measured the steady-state levels of Atg7 by Western blot in the brains of 3xTg-AD^15–18^, 3xTg-AD^2–18^ and 3xTg-AD^CTL^ mice (Fig. 7A, D). One-way ANOVA showed a significant difference in Atg7 levels between the three groups of mice (F = 46.92; p<0.001). Bonferroni's post hoc analysis showed that the levels of Atg7 were significantly higher in the brains of 3xTg-AD^15–18^ and 3xTg-AD^2–18^ compared to 3xTg-AD^CTL^ mice (p<0.01), but were not statistically different between 3xTg-AD^15–18^ and 3xTg-AD^2–18^ mice (Fig. 7A, D). Atg7 facilitates the assembly of Atg5 and Atg12, which are targeted to autophagosome vesicles [11], [13]. To determine whether the rapamycin-induced increase in Atg7 led to an increase in the Atg5/Atg12 complex, brains from 3xTg-AD^15–18^, 3xTg-AD^2–18^ and 3xTg-AD^CTL^ mice were analyzed by Western blot using an Atg5-specific antibody. Mirroring Atg7 levels, the levels of Atg5/Atg12 were significantly higher in the 3xTg-AD^15–18^ and 3xTg-AD^2–18^ compared to 3xTg-AD^CTL^ mice (Fig. 7A, E; F = 64.37; p<0.001 as calculated with one-way ANOVA and Bonferroni's post hoc test). Notably, the levels of Atg5/Atg12 were similar between 3xTg-AD^15–18^ and 3xTg-AD^2–18^ mice (Fig. 7A, E). These data show a rapamycin-mediated increase in autophagy induction, which was further confirmed by measuring the levels of LC3I and II. LC3I is an autophagy-related protein that is post-translationally modified to form LC3II during autophagy induction; LC3II is then incorporated into the membrane of the growing autophagosome [64]. Indeed, LC3II levels are routinely used to assess the levels of autophagy induction [65]. We found that while the levels of LC3I were similar among 3xTg-AD^CTL^, 3xTg-AD^15–18^, 3xTg-AD^2–18^ mice (Fig. 7A, F), the levels of LC3II were significantly increased in the two rapamycin-treated groups (Fig. 7A, G; p = 0.0001 as assessed by one-way ANOVA). Bonferroni's post hoc analysis indicated that both the 3xTg-AD^15–18^ and the 3xTg-AD^2–18^ mice were significantly different from the 3xTg-AD^CTL^ mice (p<0.001) but not from each other.

**Figure 8:**
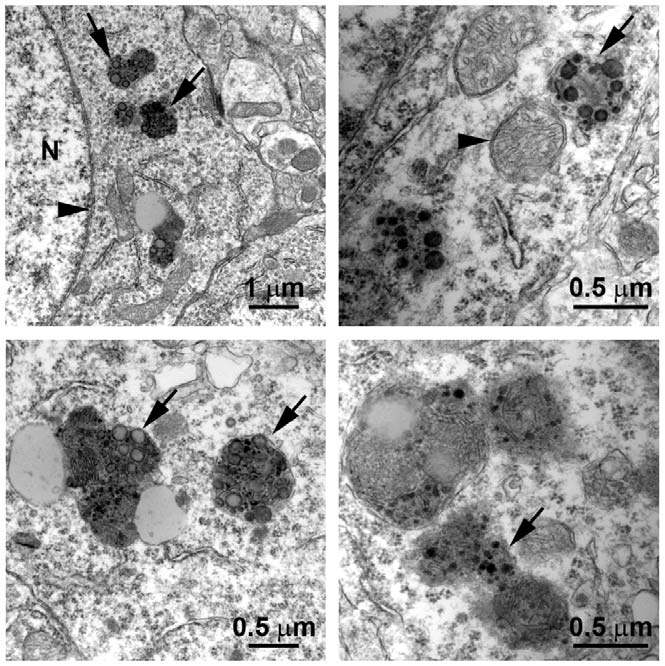
Abnormal autophagosomes in 15-month-old 3xTg-AD mice. Electron microscope sections obtained from CA1 regions of 15-month-old 3xTg-AD mice. Sections show examples of enlarged autophagosomes containing electron-dense undigested materials. Top-left panel: N = nucleus; the arrowhead points to the nuclear membrane; the arrows point to enlarged autophagosomes. Top-right panel: the arrowhead points to an autophagosome that does not contain undigested material. The arrow points to an autophagosome containing undigested material. Similar structures are also shown in the bottom two panels (arrows).

Taken together, the data presented here suggest that short- and long-term rapamycin administration induce autophagy. Additionally, the data highlight a clear dissociation between autophagy induction and Aβ clearance. Indeed, while the levels of autophagy induction were similar between 3xTg-AD^15–18^ and 3xTg-AD^2–18^ mice, only the 3xTg-AD^2–18^ mice showed a significant reduction in Aβ pathology compared to the 3xTg-AD^CTL^ mice. To start elucidating the basis of this dissociation, we conducted electron microscope studies using 15-month-old 3xTg-AD mice, the age at which the 3 months of rapamycin treatment began. We found numerous enlarged autophagosomes containing undigested electron-dense material (Fig. 8), highlighting the fact that in the 3xTg-AD^15–18^ mice, rapamycin treatment was started following the accumulation of insoluble, electron-dense aggregates. It is tempting to speculate that the electron-dense material represents irreversible aggregates, perhaps too large to be engulfed by autophagosomes or not recognized as material to be targeted for autophagic degradation. Further studies are necessary to determine why this electron-dense material is not sensitive to autophagy induction. Overall, our data show that under the conditions used here, facilitating autophagy induction prophylactically has beneficial effects on AD-like pathology; however, once plaques and tangles are well established, increasing autophagy induction is not sufficient to rescue AD-like pathology and the associated cognitive deficits.

## Discussion

Plaques and tangles are the two hallmark lesions of AD. Recently, more attention has been focused on soluble Aβ and tau as strong evidence shows that the build-up of these two species plays a critical role in AD pathogenesis [66], [67]. That is not to say, however, that mature plaques and tangles do not contribute to the cognitive deficits associated with AD. In fact, elegant electrophysiological and imaging studies show that plaques directly alter calcium signaling and physiological neuronal firing, as well as cause structural alterations in the brains of AD transgenic mice [3], [4]. Thus, when evaluating a possible therapeutic compound, it is critical to assess its effects not only on soluble Aβ and tau but on mature plaques and tangles as well. We, and others, have previously shown that rapamycin reduces soluble Aβ and tau pathology and the associated early cognitive deficits in 6-month-old transgenic mice [29], [32]. Here we significantly extend our previous findings and use a pharmacological approach to increase autophagy and determine the effects on plaques and tangles formation and/or plaques and tangles degradation. We found that only when administered prophylactically, throughout life, does rapamycin increase autophagy induction and reduce the formation of plaques and tangles, likely by increasing soluble Aβ and tau turnover. In contrast, if rapamycin is given to 15-month-old mice, after plaques and tangles are well-established throughout the brain, no changes in soluble or insoluble Aβ and tau levels are detected; consequently, cognitive deficits remain unchanged.

The role of autophagy in AD is controversial. For example, Nixon and colleagues have shown that in AD brains there is accumulation of autophagosomes [68]. Additionally, they showed that autophagosomes may be another source of Aβ generation, suggesting that interventions aimed at further increasing autophagy induction in AD may actually exacerbate the Aβ pathology [69]. In contrast, Wyss-Coray and colleagues have shown that increasing autophagy induction decreases Aβ pathology in an animal model of AD [70]. Data in apparent contradiction to each other have been also reported by others [5], [29], [32], [71], [72], [73], [74], [75]. Our data are compatible with both views as we show that increasing autophagy induction prior to the development of AD-like pathology in the 3xTg-AD mice reduces the levels of soluble Aβ and tau and the formation of thioflavin-positive plaques. In contrast, we show that if autophagy is induced after mature plaques and tangles are formed, no changes in Aβ, tau or cognitive deficits are detected. We suggest that increasing autophagy induction may be a valid therapeutic strategy for AD if the intervention occurs early in the development of the disease. In contrast, once the neuropathology is well-established, increasing autophagy induction alone may not be sufficient to ameliorate the neuropathological phenotype and different approaches should be considered. Toward this end, recently it has been shown that reversing autophagy dysfunction by increasing lysosomal function, is a good strategy in AD [76].

Rapamycin is a product of the bacterium *Streptomyces hygroscopicus*, first discovered in soil from Easter Island [77]. It is currently used in the clinic as an immunosuppressant to prevent organ rejection during transplant [78]. Additionally, because of its anti-proliferative properties, rapamycin is also being used in clinical trials for cancer treatment [79]. In aging and age-related disorders, rapamycin has been shown to increase lifespan and healthspan in mice [16]. Additionally, a recent report showed that rapamycin reverses the cellular phenotype associated with Hutchinson-Gilford progeria syndrome, a lethal genetic disorder characterized by premature aging [80]. Here we show that life-long rapamycin treatment has no overt negative consequences on health; indeed, mice on rapamycin gain weight at the same pace as mice on the control diet. Notably, we also show that rapamycin acts directly on brain mTOR signaling, clearly suggesting that rapamycin crosses the blood-brain barrier. This is consistent with previous reports showing that rapamycin crosses the blood brain barrier in humans and various experimental models [52], [57], [58], [59], [60]. Specifically, Cloughesy and colleagues directly measured rapamycin levels in brain tumors extracted from patients affected by neuroblastoma after one week of daily rapamycin administration and showed that rapamycin was present in the brain tissue, clearly indicating that rapamycin crossed the blood-brain barrier [57].

We have previously shown that in the 3xTg-AD mice tau pathology is highly dependent on Aβ accumulation [31], [81], [82], [83]. For example, we have shown that intrahippocampal injections of anti-Aβ antibodies were sufficient to reduce tau pathology [81]. Additionally, we showed that genetically preventing Aβ accumulation was sufficient to greatly delay tau accumulation, even though the tau transgene levels remained unaltered [31]. Here we show that rapamycin reduces the hyperactive mTOR signaling in the 3xTg-AD mice and decreases Aβ and tau pathology. However, it remains to be established whether the changes in tau pathology are directly due to an interaction between mTOR signaling and tau or are simply due to a decrease in Aβ pathology. Indeed, a link between tau and mTOR has been proposed by different laboratories. For example, PI3K/mTOR signaling regulates tau phosphorylation [84] and TOR activation enhances tau-induced neurodegeneration in a *Drosophila* model of tauopathies [85]. Further strengthening the mTOR/tau link is the data from studies of AD brains showing that mTOR signaling is selectively increased in neurons predicted to develop NFTs and that such an increase correlates with tau phosphorylation [23], [27], [86], [87]. This evidence has led to the hypothesis that the chronic increase in mTOR function that take place during aging may facilitate the development of tau pathology [86]. Further studies are necessary to assess whether the rapamycin-mediated effects on tau pathology are due to a direct link between tau and mTOR or are due indirectly to a decrease in Aβ levels.

In conclusion, pharmacologically increasing autophagy may be a valid therapeutic approach to prevent or treat AD and other age-related neurodegenerative disorders characterized by the accumulation of misfolded proteins [88]. Toward this end, it has been shown that increasing autophagy by rapamycin administration decreases alpha-synuclein- and huntingtin-related neuropathologies in animal models of Parkinson and Huntington diseases, respectively [89], [90]. Finally, considering the role of aging in AD [91], in theory, agents capable of delaying aging should also have beneficial effects on AD phenotype. Because of its anti-aging properties and its effects on clearing protein deposits, rapamycin should be considered in future clinical trials for age-dependent neurodegenerative disorders.

## Materials and Methods

### Mice and rapamycin administration

The mice used in these studies have been previously described [30]. Both 3xTg-AD and NonTg mice used in these studies were on a mixed C57BL6/129svj background. Microencapsulated rapamycin was added at a concentration of 14 mg/kg to mouse chow, as described previously [16]. Food containing empty microcapsules, i.e., the microencapsulation material without the drug, was used as the control diet. During the treatment, mice were given *ad libitum* access to water and the rapamycin or control diet. Body weight was measured before the beginning of the treatment and monthly thereafter. All animal procedures were approved by The Institutional Animal Care and Use Committee of The University of Texas Health Science Center at San Antonio; Animal protocol number 11071x, approved on June 27, 2011.

### Behavioral tests

Morris water maze tests were conducted in a circular tank of 1.5 meters in diameter, located in a room with several extra maze cues as described previously [92]. Briefly, the platform (14 cm in diameter) location was kept constant for each mouse during training and was 1.5 cm beneath the surface of the water, which was maintained at 25°C throughout the duration of the testing. Mice received 4 trials a day and were alternated among 4 pseudorandom starting points for 5 consecutive days. If a mouse failed to find the platform within 60 seconds, it was guided to the platform by the researcher and kept there for 20 seconds. The inter-trial interval was 25 sec, during which time each mouse was returned to its home cage. Probe trials were conducted 24 hours after the last training trial. During the probe trials, the platform was removed and mice were free to swim in the tank for 60 seconds. The training and probe trials were recorded by a video camera mounted on the ceiling, and data were analyzed using the EthoVisioXT tracking system.

The object recognition test was conducted in a clear Plexiglas box (40×40 cm) and was recorded with a video camera mounted above the testing box. Mice were left free to explore two objects for 5 minutes in the same arena used for open-field activity. After a 10-minute delay, where the mice were returned to the home cage, the mice were returned to the arena where one of the two objects was replaced with a new object. The percentage of time spent exploring the new object was measured with EthoVision.

### Protein extraction, Western blot, and ELISA

Mice were sacrificed by CO_2_ asphyxiation and their brains extracted and cut in-half sagittally. For immunohistochemical analysis, one-half of the brain was dropped-fixed in 4% paraformaldehyde in PBS for 48 hours and then transferred in 0.02% sodium azide in PBS until slicing. The other half was frozen in dry ice for biochemical analysis. Frozen brains were homogenized in a solution of tissue protein extraction reagent (T-PER, Pierce, Rockford, IL) containing 0.7 mg/ml Pepstatin A supplemented with a complete Mini protease inhibitor tablet (Roche, Switzerland) and phosphatase inhibitors (Invitrogen, Carlsbad, CA). The homogenized mixes were briefly sonicated to sheer the DNA and centrifuged at 4°C for 1 hour at 100,000 g. The supernatant was stored as the soluble fraction. The pellet was re-homogenized in 70% formic acid and centrifuged as above. The supernatant was stored as the insoluble fraction.

For Western blot analyses as described in [93], proteins from the soluble fraction were resolved by 10% Bis-Tris SDS/PAGE (Invitrogen, Carlsbad, CA) under reducing conditions and transferred to a nitrocellulose membrane. The membrane was incubated in a 5% solution of non-fat milk for 1 hour at 20°C. After overnight incubation at 4°C with primary antibody, the blots were washed in Tween 20-TBS (T-TBS) (0.02% Tween 20, 100 mM Tris pH 7.5; 150 nM NaCl) for 20 minutes and incubated at 20°C with secondary antibody. The blots were washed in T-TBS for 20 minutes and incubated for 5 minutes with Super Signal (Pierce, Rockford, IL), washed again and exposed. Aβ40 and Aβ42 levels were measured from the soluble and insoluble fractions using a sandwich ELISA protocol as described in [94].

### Immunohistochemistry

For immunohistochemical analysis, 50 µm thick sections were obtained using a Leica vibratome slicing system and sections were stored at 4°C in 0.02% sodium azide in PBS. To quench the endogenous peroxidase activity, free-floating sections were incubated for 30 minutes in H_2_O_2_. For the Aβ staining, sections were subsequently incubated in 90% formic acid for 7 minutes to expose the epitope. The appropriate primary antibody was applied and sections were incubated overnight at 4°C. After removing the primary antibody in excess, sections were incubated in the appropriate secondary antibody for 1 hour at 20°C. After a final wash of 20 minutes, sections were developed with diaminobenzidine (DAB) substrate using the avidin-biotin horseradish peroxidase system (Vector Labs, Burlingame, CA). Images were obtained with a digital Zeiss camera and analyzed with ImageJ software.

### Electron microscope experiments

These experiments were conducted in the Pathology Electron Microscopy Facility of the University of Texas Health Science Center at San Antonio using standard techniques as described previously [44].

### Statistical analyses

Statistical analyses used here were detailed in [95]. Briefly, tests were conducted using multifactor analysis of variance, following by the Bonferoni's multiple comparison test to determine individual difference among groups. Calculations were performed using GraphPad Prism version 3.00 for Windows, GraphPad Software, San Diego California USA, www.graphpad.com.
